# Genetic Mechanisms of the Devious Intruder *Candidatus Liberibacter* in *Citrus*

**DOI:** 10.3389/fpls.2017.00904

**Published:** 2017-05-31

**Authors:** Federico Martinelli, Abhaya M. Dandekar

**Affiliations:** ^1^Dipartimento di Scienze Agrarie Alimentari e Forestali, Università degli Studi di PalermoPalermo, Italy; ^2^Plant Sciences Department, University of California, DavisDavis, CA, United States

**Keywords:** carbohydrate, *Candidatus liberibacter*, *citrus*, detoxificant pathways, jasmonic acid, Huanglongbing, salicylic acid

## Huanglongbing is the most threatening disease in *Citrus*

Citrus Huanglongbing (HLB), or citrus greening disease, is the most important, destructive and dangerous disease of citrus in the world (Bové, 2006). It is caused by endogenous, sieve vascular-restricted liberibacters (*Candidatus liberibacter;* Ca.L.). Ca.L. can infect most citrus cultivars, species and hybrids and even some citrus relatives. HLB symptoms are similar in all three types: (Ca. L. *asiaticus*, Ca. L. *africanus*, and Ca. L. *americanus*). The affected leaves develop a pattern of yellow and green areas lacking clear limits between the colors, giving a “blotchy mottle” appearance (McClean and Schwarz, 1970). Ca.L. is mainly present in leaf veins and petioles; however, it is has also been detected in bark, roots, and fruit peel. The disease causes fruit malformation and altered phenotype. Fruits are colored green to yellow/orange, usually small, asymmetric, and lopsided, with a bent fruit axis and small or aborted seeds. Symptoms are similar to those of zinc deficiency, although Huanglongbing disease causes asymmetric leaf yellowing. After symptoms appear, leaves generally drop and twig dieback occurs. Another typical effect of HLB infection is excessive fruit drop (Bové, 2006). Over 100 million HLB-infected citrus trees have been destroyed to limit disease spread since HLB was first recognized (Halbert and Manjunath, 2004).

Diseased trees decline, their yield is reduced, and fruit quality is impaired greatly. Although brassinosteroids, small host-response modulating molecules, and a mixture of antibiotic compounds can improve tree health and create interesting, beneficial molecular responses (Puttamuk et al., 2014; Canales et al., 2016; Martinelli et al., 2016a), there is no sustainable, technically feasible therapy for affected trees. The lack of cure and relatively fast progression of the disease in orchards made it vital to investigate HLB in depth, at the molecular level, to better understand the pathways used by Ca.L. to infect and grow in trees and its tissue-specific effects and symptoms (Dandekar et al., 2010; Martinelli et al., 2016b). Although recent studies provided insight into the possible pathogenetic mechanisms (Gardner et al., 2016), it is still not clear how the pathogen causes the well-known symptoms.

Extensive transcriptomic studies comparing healthy, infected (at different stages), slightly resistant, and tolerant genotypes suggest that the pathogen modulates key genes involved in carbohydrate metabolism (Albrecht and Bowman, 2008; Kim et al., 2009; Mafra et al., 2013), drastically affecting the flow of nutrients throughout the tree, with consequent source-sink disruption that decreases fruit production and quality and ultimately kills the tree (Martinelli et al., 2012, 2013).

## Metabolic disorder of carbohydrate metabolism

Significant HLB-modulation of key genes involved in carbohydrate biosynthesis and metabolism, especially starch biosynthesis, has been described (Albrecht and Bowman, 2008) and subsequently confirmed (Kim et al., 2009). Increased starch is linked to a diverse response to Ca.L. infection between suceptible and tolerant genotypes (Fan et al., 2013). The enhanced accumulation of starch is probably due to the stimulated entrance of glucose into the pathway linked with the upregulation of glucose-phosphate transport (GPT2) in leaf tissues. The key role of this protein is corroborated by the absence of HLB-regulation of the gene in symptomatic fruit tissues where starch is not accumulated.

A meta-analysis of transcriptomic data from susceptible and resistant genotypes showed that carbohydrate metabolism and biological process associated with biotic stress response were key pathways affecting HLB progression of symptoms (Rawat et al., 2015). Phloem cell disruption and increased starch are associated with a diverse response to Ca.L. infection among genotypes with different susceptibility (Fan et al., 2013). The transcriptomes of fruit peel from diseased (three different stages) and healthy fruits were analyzed in detail (Martinelli et al., 2012). Differentially affected genes and pathways were identified using an integrated approach of principal component analysis, gene and pathway enrichment analysis, predicted protein-protein interaction network analysis, and extensive qRT-PCR validation of citrus genes. HLB affected transcription of genes involved in light reactions of photosynthesis, ATP synthesis, protein degradation, and protein misfolding processes. The induction of photosynthesis genes is consistent with the green color of symptomatic fruits. Infected trees also had increased source-sink communication (hydrolases or sugar/nutrient starvation), sucrose and starch metabolism, and drastically altered hormonal crosstalk and signaling (cytokinins and gibberellins repressed and ethylene induced). There were significant differences in transcriptomic changes among organs (stems and root tissues) (Aritua et al., 2013).

These findings lead to the hypothesis that pathogen colonization of a citrus tree provokes disruption of source-sink communications. Because these changes are observed at an early, asymptomatic stage, before the phloem plug is observed, these molecular changes may be more involved in causing the disease than an effect of it. The important role of carbohydrates in promoting Ca.L. growth in infected trees is also confirmed at the metabolic level. Metabolomic studies conducted on phloem sap showed that the concentration of mono- and disaccharides, sugar alcohols, and sugars in the phloem affects Ca.L. growth in phloem (Slisz et al., 2012; Albrecht et al., 2016; Killiny, 2016).

Although phloem necrosis contributes to impaired host nutritional transport functions and source-sink communications, upregulation of key genes involved in glucose transport, sucrose metabolism and starch biosynthesis occurred before HLB symptoms appear (Martinelli et al., 2013, 2015). A predicted protein—protein interaction network identified HLB-regulated genes for sugar transporters with key roles in overall plant responses.

**Figure 1:**
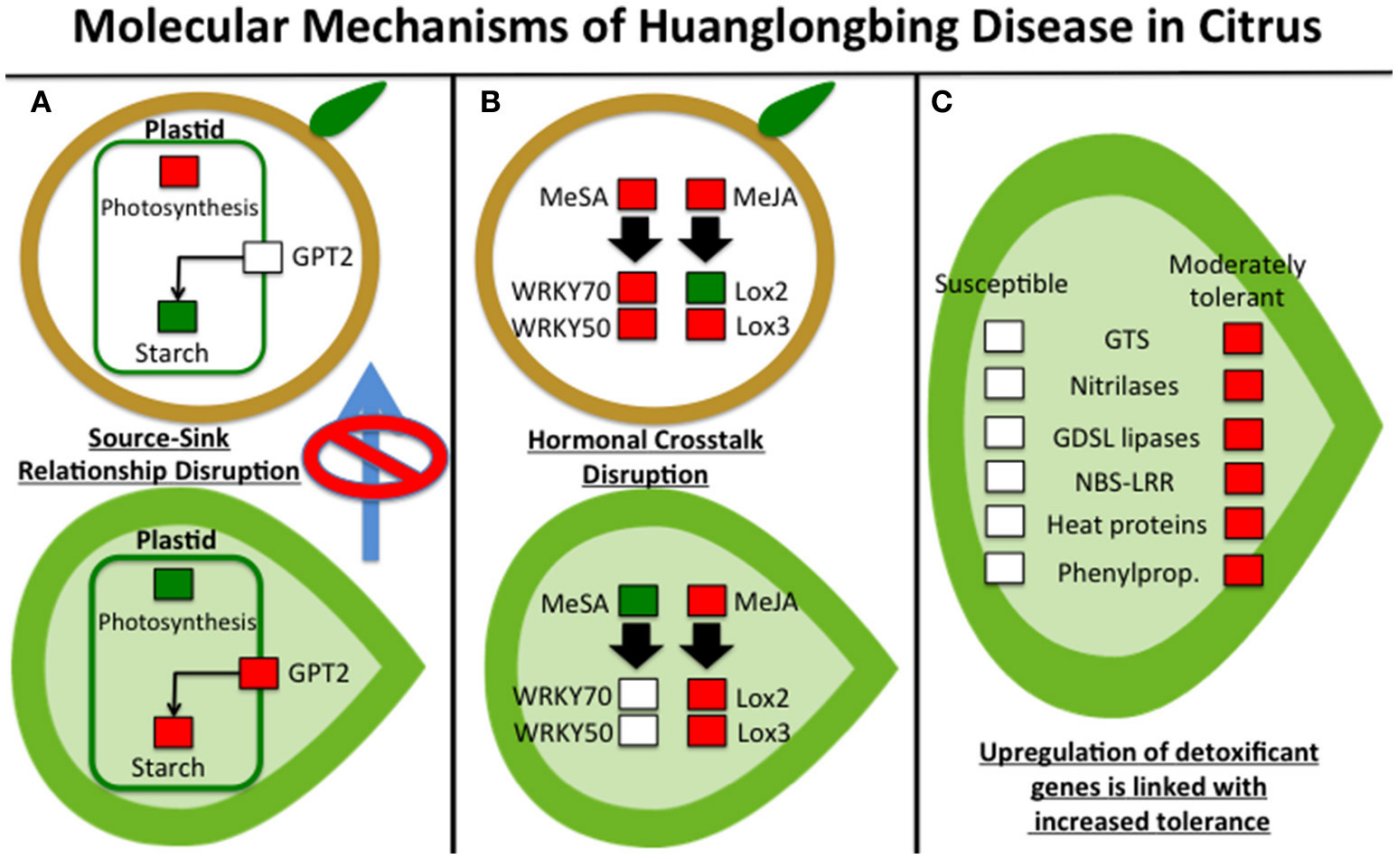
The three main molecular mechanisms driving symptom progression of Huanglongbing disease in citrus: **(A)** source-sink disruption due to starch accumulation in leaves, stimulated by upregulation of *glucose-phosphate-transport2* (GPT) and the induction of genes involved in photosynthetic reactions in fruits; (B) non-beneficial modulation of jasmonic acid-salicylic crosstalk that is not correlated with the different pathogen concentration in leaf and fruit tissues; and (C) modulated expression of detoxifying proteins linked with different susceptibility of citrus genotypes. Red color means “upregulated,” green means “downregulated” while white means “not-regulated.” Genes not mentioned in the text were: NBS-LRR (nucleotide binding site leucine rich repeats), lox2, and lox3 (lipoxygenase2 and 3), GTS (glutathione-S-transferases).

We suggest that upregulation of invertases blocks sucrose export and may decrease photosynthesis and stunt growth, with subsequent yellowing of leaves. The leaves (source) showed enhanced expression of invertase in addition to the fruit (sink). Glycolysis and sucrose metabolism were upregulated due to disrupted source-sink transport. Differential expression of key genes involved in sucrose and starch metabolism in Ca.L.-infected citrus fruit may affect the osmotic potential and induce plasmolysis, altering the ripening process and producing typical HLB symptoms. This leads to subsequent metabolic dysfunction. Increased photosynthesis also increased ROS (reactive oxygen species), causing oxidative stress. HLB-regulated genes (*glucose-phosphate-transporter, invertase*, and starch-related genes) determine the disruption of the source-sink relationship. In infected leaves, transcriptomic changes were observed in light reaction genes (downregulation), sucrose metabolism (upregulation), and starch biosynthesis (upregulation; Figure 1). In parallel, symptomatic fruits over-expressed genes involved in photosynthesis and sucrose and raffinose metabolism, while downregulating starch biosynthesis (Martinelli et al., 2012). The visualization of gene regulatory networks affected by Ca.L. in fruits and leaves at different developmental stages clearly showed a source-sink shift (Martinelli et al., 2013).

## Altered hormonal crosstalk diverts appropriate citrus immune responses

Other critical transcriptomic changes induced by Ca.L. affect hormonal crosstalk-mediated immune responses. Ca.L. infection induces genes involved salicylic acid and jasmonic acid signaling (methyl-salicylate (MeSA) and methyl-jasmonate (MeJA), but not in a beneficial way and not in the correct tissues (Figure 1). Systemic acquired responses were inadequately activated in young leaves, the location where most new infections occur (Martinelli et al., 2013). Ca.L. infection induces salicylic acid and jasmonic acid production, increasing expression of WRKY family proteins and transcription factors. However, expression of WRKYs (including *WRKY70* and *WRKY50*) was greater in fruits than in leaves.

Ca.L. induces biotrophic behavior instead of necrotrophic effects in colonized host tissues. Increased systemic acquired response should be greater than jasmonic acid-mediated signaling. Although jasmonic acid-mediated signaling is triggered by the insect vector, SAR (systemic acquired resistance) responses should be much more pronounced, especially in young leaves, where infections typically occur. We hypothesize that the disease interferes with the hormone-driven crosstalk network, drastically affecting plant immune responses. The resulting unsatisfactory plant response to pathogen growth leaves it free to colonize the tissue and disrupt the source-sink relationship. Ca.L. alters hormone crosstalk, resulting in a weak, ineffective SAR response for a biotroph pathogen such as Ca.L. The importance of SAR in inducing resistance to this type of pathogens was shown in mandarin at 2 years from infection (Xu et al., 2015) and in transgenic experiments that overexpresses a citrus ortholog of *NDR1* (Non-race-specific disease resistance 1) in *Arabidopsis* (Lu et al., 2013).

## The modulation of antioxidant pathways

The upregulation of genes involved in oxidoreductase reactions validated the theory that HLB causes oxidative stress. In a recent proteomic study, upregulation of proteins involved in detoxification pathways was linked to increased tolerance to Huanglongbing disease (Martinelli et al., 2016c; Figure 1). Nitrilases, GDSL lipases and the glutathione-S-transferases GST30, GST18, and GSTF9 were upregulated in Volkameriana (a moderately tolerant genotype), but not in Navel orange (a highly susceptible genotype). These proteins have a role in radical ion detoxification. We suggest that induction of proteins involved in xenobiotic responses is strongly associated with increased tolerance to HLB. Our data confimed previous proteomic findings regarding proteins involved in the induction and detoxification pathways in infected leaf tissues (Nwugo et al., 2013). Heat shock proteins decreased in HLB-infected trees, especially HSP70 and HSP82, which stabilize proteins and facilitate refolding of proteins that have been denatured (Martinelli et al., 2012). These data were confirmed by comparative proteomic analysis conducted after heat treatment at 40°C for 6 days (Nwugo et al., 2016). Two citrus genotypes showed increased pathogenesis-related proteins after Ca. L. infection (Martinelli et al., 2016c). However, the greater tolerance of Volkameriana over Navel orange was linked to greater activation of glutathione-S-transferases and upregulation of enzymes involved in biosynthesis of peroxiredoxins, Cu/Zn superoxide dismutases and 2Fe-2S ferredoxin-like proteins. We suggest that glutathione-S-transferases are important modulators of citrus tolerance to HLB disease. Stress-inducible glutathione-S-transferases can neutralize dangerous compounds provoked by oxidative damage (Albrecht and Bowman, 2008). An important factor contributing to citrus suceptibility may be the failure to rapidly detoxify the reactive oxygen species produced by Ca.L.

## Conclusions

HLB symptom progression may result from three types of dysfunction occurring in Ca.L.-infected citrus: (1) a carbohydrate disorder linked to disruption of the source-sink relationship, (2) perturbation of hormonal crosstalk involved in plant immune responses (JA-SA signaling crosstalk), and (3) changes in the rapid activation of detoxifying pathways (particularly GSTs). The development of innovative short- or long-term biotechnological tools that allow beneficial modulation of these three pathways will help increase *Citrus* tolerance to this devastating disease.

## Author contributions

All authors listed, have made substantial, direct and intellectual contribution to the work, and approved it for publication. FM mainly wrote the article. AMD and FM conceived, designed and developed the concepts expressed in this article.

### Conflict of interest statement

The authors declare that the research was conducted in the absence of any commercial or financial relationships that could be construed as a potential conflict of interest.
